# Effect of bolting on roadway support in extremely weak rock

**DOI:** 10.1186/s40064-016-3031-6

**Published:** 2016-08-17

**Authors:** Qinghai Li, Weiping Shi, Zhongcheng Qin

**Affiliations:** 1 State Key Laboratory of Mining Disaster Prevention and Control Co-founded by Shandong Province and the Ministry of Science and Technology, Shandong University of Science and Technology, Qingdao, 266590 China; 2 College of Geomatics, Shandong University of Science and Technology, Qingdao, 266590 China

**Keywords:** Physical simulation, Roadway supporting, Bolting, Extremely weak rock, Reinforcing coefficient

## Abstract

In mine roadway support operations, floor bolting not only played a role in floor heave control, but also in reinforcing roof and its two sides. Correspondingly, bolting of roof and two sides also played a part in floor heave control. To quantify the effect of such bolting, based on roadway support in extremely weak rock, three physical models were produced and tested in laboratory. Through comparison of their displacements in three physical simulation experiments, the reinforcing effect of bolting in extremely weak rock roadways was quantified. Reinforcing coefficients was defined as displacement ratio between original support and new support regime. Results indicated that the reinforcing coefficients, for bolting of roof and its two sides, on floor, two sides, and roof reached 2.18, 3.56, and 1.81 respectively. The reinforcing coefficients for floor bolting on floor, two sides, and roof reached 3.06, 2.34, and 1.39 respectively. So in this extremely weak rock, the surrounding rock should be considered as an integral structure in any support operation: this allows for better local strength improvement, and provided future design guidance.

## Background

Roadway control in soft rock is a problem in many mines (Bilir 2011; Ghiasi et al. 2012; Serafeimidis and Anagnostou 2013; Thomas et al. 2013). When roadways are excavated, pressure is redistributed. Under this mining induced stress, roadways in soft rock undergo large, continuous deformation as they are excavated.

Three rock mass classification systems, the rock mass rating (RMR), the rock mass strength (RMS), and the slope mass rating (SMR) have been widely applied to areas of hard rock and weak rock in civil engineering. Brook and Hutchinson (2008) indicated that the relative weightings of the different parameters within the RMR, RMS, and SMR classification schemes would need modifying for weak rock masses, but the precise details of this were difficult to determine. In mine, based on RMS, content of mudstone, features of joint planes, and the mechanism of plastic deformation, weak rock was classified into four types (He et al. 2002): extremely weak rock (swelling soft rock), high-stress soft rock, jointed soft rock and mixed soft rock.

Extremely weak rock, with a lower compressive strength, a lower tensile strength, a reduced apparent cohesion and poor cementation, always contains swelling (smectite) clay minerals. This rock was vulnerable to weathering, which became more serious when in contact with water. When roadways are excavated in this rock, even in lower stress conditions (<25 MPa), the cross-sections can be disrupted and the plastic failure zone in surrounding rock always extends significantly. Indeed, mudstone in Cretaceous strata always exhibits such behaviour.

While investigating the mechanisms underpinning swelling behaviour in such soft rock, Christoph et al. (2011), Luciano and Eduardo (2012) indicated that the dissolution of anhydrite and precipitation of gypsum occurred upon the uptake of water. Einstein (1996) indicated that, in argillaceous rocks, swelling is caused by one, or a combination of, intracrystalline, osmotic, and mechanical effects. In a tunnel, Butscher et al. (2011a, b) concluded that excavation-induced groundwater inflow into anhydritic layers and caused rock swelling. Pejon and Zuquette (2002) and Moosavi et al. (2006) indicated that deformation of a roadway in swelling soft rock depended on mineralogy, lithology, ground characteristics, hydrology, stress state, and weathering conditions. In addition to this, using an artificial neural network, Doostmohammadi et al. (2008) investigated the swelling potential of mudstone. Lo and Hefny (1996) developed rheological models, which could be used to predict the swelling effects on a circular tunnel.

High-stress soft rock, always with less argillaceous components, demonstrates a high compressive strength, a high tensile strength and a good cohesion. This rock can be deformed obviously under high stress conditions (>25 MPa). High stresses were the main factor causing roadway deformation. For the same strength of surrounding rock, when under different stress conditions, the roadway may demonstrate different deformation patterns. In low-stress conditions, the roadway was stable and no obvious deformation occurred; however, when under high stress conditions, the roadway may demonstrate rheological behaviour, such as soft rock deformation: the deformation can be large, and thus causes difficulties in roadway control. Strata at 1000 m below ground have always presented such characteristics.

Jointed soft rock, always with less and even no argillaceous components, contains many joints in rock body. In such soft rock, each block demonstrates high strength. But the whole body exhibits soft rock characteristic and can be deformed largely, which is induced by joints’ plane slipage and dilatation, under mining induced stress.

Mixed soft rock is the combination of above three soft rocks, including high stress-swelling soft rock, high stress-jointed soft rock and high stress-swelling-jointed soft rock.

To control soft rock deformation, numerous studies have advocated various support strategies and tested in situ.

In swelling soft rock, support measures include either the application of a strong, rigid supporting formwork to limit deformation, or allowing floor heave to release swelling pressures, or a combination of both (Christoph et al. 2011). In Canada, a tunnel situated in the Queenston Formation, South Ontario, was supported by a double shell lining system, which included an initial lining of shotcrete, steel ribs, and rock dowels, and a final lining of waterproofing membrane and cast-in-place concrete (Ansgar and Thomas 2010). In the T13 tunnel, Ankara–Istanbul High-Speed Train Project, a heavier, non-deformable support system was applied (Aksoy et al. 2012). In tertiary soft rock roadway in Liuhai coal mine, China (Yang et al. 2015), bolt-mesh-cable and double-layer-truss supports were used to control the large rheological deformation. Shen (2014) proposed a support system, which included an optimal cable/bolt arrangement, full length grouting, and high-load pre-tensioning of bolts and cables.

In high-stress soft rock, according to Chang et al. (2013), use of a double layer of 40 U-shape steel sets, cables, resin bolts in both ribs and roof, and swellex bolts in floor were used in roadway support works. Sun et al. (2014) indicated that the asymmetric coupling support of bolt net spray, with anchor cables, and floor bolts could enhance the stability of deep roadway. Li et al. (2015) proposed a combined support system including: high-toughness sealing layer, hollow grouting cables, and full-length anchor bolts. Li et al. (2014) recommended a coupling support of double yielding shells, which can control roadway deformation in high stress soft rock.

In most control measures, bolting in surrounding rock was used to better effect (Guo et al. 2012; Wang et al. 2012; Meng et al. 2013; Kang et al. 2015; Sun and Wang 2011; Wang et al. 2015; Yuan et al. 2014; Yu et al. 2015; Zhao et al. 2015). Since the surrounding rock was treated as a whole structure, bolts in the floor not only played a role in controlling floor heave, but also played a role in reinforcing roof and two sides. Correspondingly, bolts in roof and two sides not only played a part in reinforcing the roof and the two sides, but also played a role in controlling floor heave. To quantify the effect of bolting, based on extremely weak rock in the No. 1 mine of Chagannuoer, three physical simulation experiments were conducted. Through displacement comparison in three experiments, we analysed the quantitative effect of boltig in the roof and two sides, and the effect of floor bolting in reinforcing the surrounding rock, which would provide guidance for roadway support in future.

## Geology and ground stresses

The No. 1 mine of Chagannuoer (NMC), located in Xilin Gol League, Inner Mongolia, China, with production capacity of 8.0 Mt/a, was under construction. Within the scope of mining area, all coal seams were stable with a near-horizontal dip angle. There was a slight syncline (20 km in length, 4–10 km in width) in the mining area. In general, the geological structure in this area was simple.

**Fig. 1:**
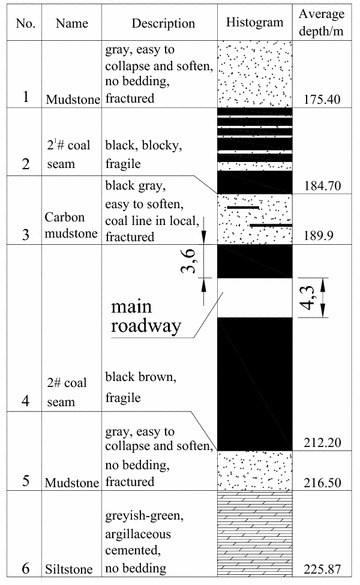
Main roadway histogram

**Fig. 2:**
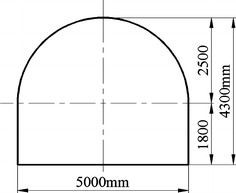
Roadway section and size

In NMC, the No. 2 coal seam, at 22.3 m thickness on average, contained lignite and lay between Cretaceous and Jurassic strata, was the main mining seam and was burried at a depth of 212.2 m. Part of the main roadway histogram is shown in Fig. 1. Roof and floor strata of the No. 2 coal seam were primarily mudstone and carbon mudstone with extremely low strengths (Table 1): these were lower than the strength of the coal seam and were in a loose, fractured state. In the mudstone, the amount of swelling clay minerals (montmorillonite) reached 49.7 %. When interacting with water, the roof and floor became mud and swelled rapidly. In mine design, considering lower strength of roof and floor, the main roadway was placed in the No. 2 coal seam (Fig. 1). The main roadway was designed as a straight wall-semicircular arch (Fig. 2). The height of its straight wall was 1800 mm and the diameter of the semicircular arch was 5000 mm. The net cross-sectional area of the roadway was 18.82 m^2^.

**Table 1:** Mechanical parameters of the strata

Name	Bulk density (average)/(t/m^3^)	Porosity (%)	Compressive strength (average)/MPa	Tensile strength (average)/MPa	Apparent cohesion (average)/MPa	Internal friction angle/(°)	RMR	GSI	*Q*
Mudstone/carbon mudstone	1.66–2.14 (2.0)	26.20–54.11	0.12–5.12 (1.71)	0.06–0.39 (0.12)	0.24–0.39 (0.31)	25.16–29.01	16	10	0.0125
Siltstone	2.01–2.65 (2.2)	17.48–27.13	0.64–8.00 (2.26)	0.07–0.61 (0.34)	0.23–0.50 (0.38)	13.70–25.50	13	8	0.0105
Coal	1.16–1.35 (1.3)	25.40–45.72	1.18–9.44 (3.44)	0.35–1.13 (0.56)	0.42–1.02 (0.66)	27.05–32.03	22	18	0.0167

**Fig. 3:**
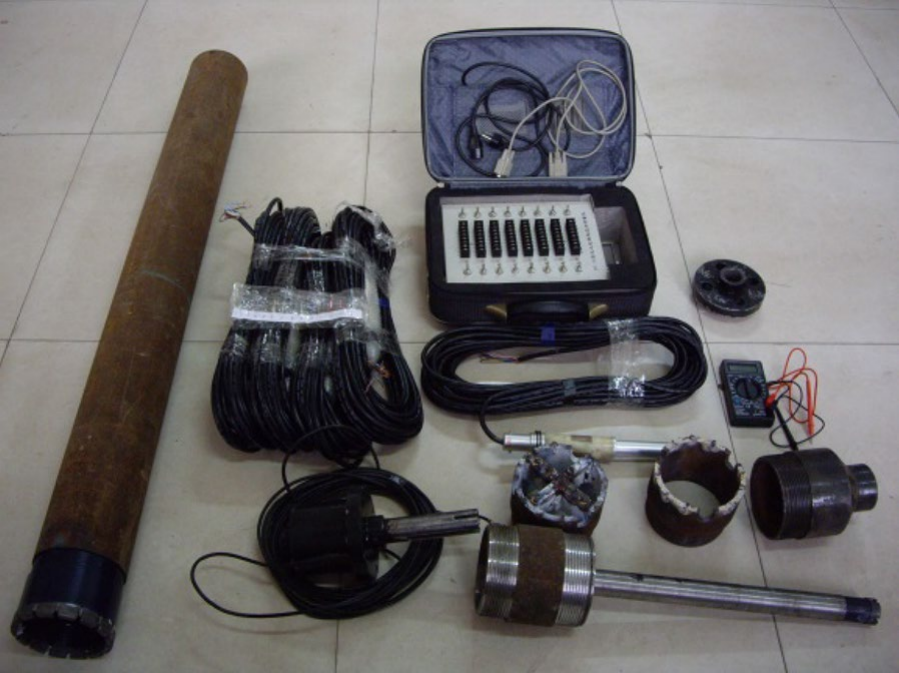
Associated equipments of overcoring method in ground stress measurement

**Fig. 4:**
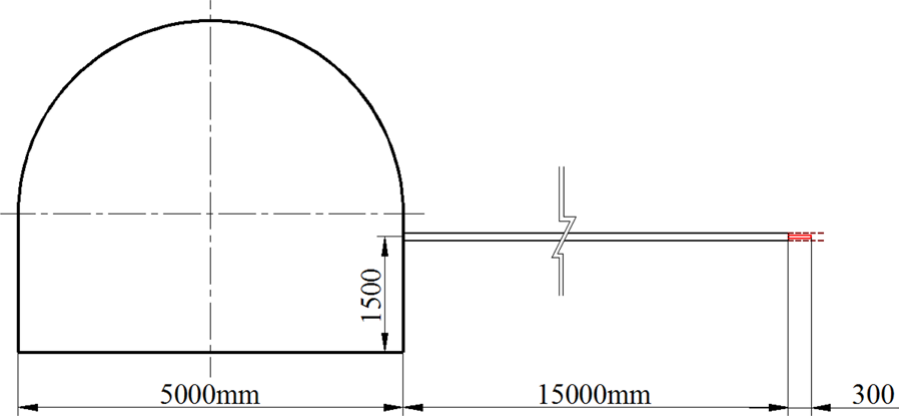
Holes layout during ground stress measurement

To find the orientation and magnitude of the ground stresses, stress measurements were conducted in three locations (A, B, and C). The overcoring method was employed in measurement. Associated equipments were shown in Fig. 3. At each location, measurements were taken in sidewall. Big hole, with 100 mm diameter and 15,000 mm depth, was drilled out 1500 mm above floor. Small hole, with same center as big hole and diameter 40 mm, was drilled out at the end of big hole (Fig. 4). Instrument was installed in the small hole. Data was acquired while instrument reliefing from body rock.

**Fig. 5:**
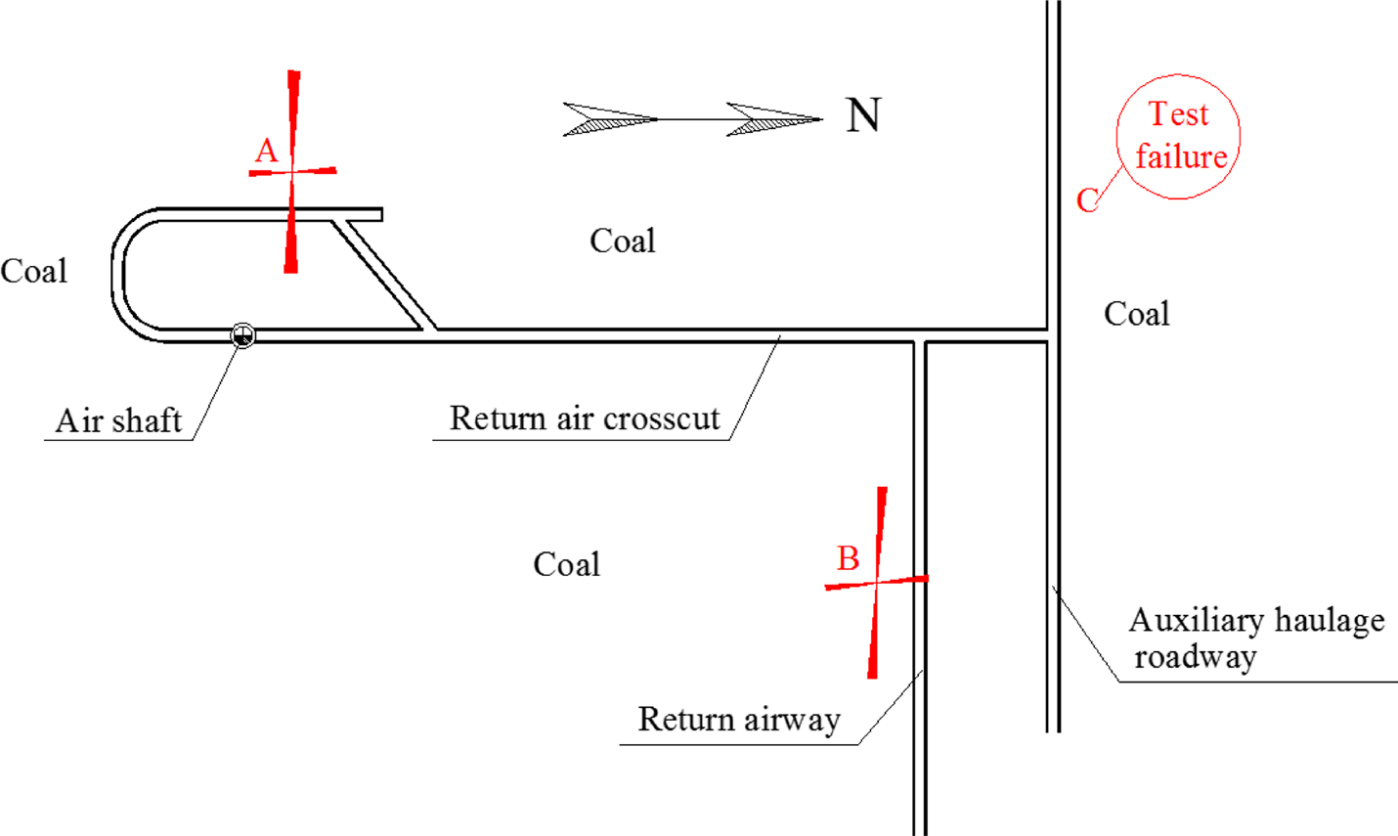
Locations of measurement sites and the orientations and magnitudes of measured horizontal stresses

The results from successful measurements at locations A and B were summarised in Table 2 and shown in Fig. 5 (procedural errors meant that no measurements were got at C). From the results it was found that the maximum principal horizontal stress lay approximately in an East–West direction. The simple geological conditions mean that *σ*_*h*max_ was closer in A and B. The ratios between *σ*_*h*max_ and *σ*_*v*_ were almost the same (1.76 and 1.78).

**Table 2:** Ground stress measurements

Location	*σ*_*h*max_ (MPa)		*σ*_*h*min_ (MPa)		*σ*_*v*_ (MPa)	*σ*_*h*max_/*σ*_*v*_
	Magnitudes/MPa	Azimuth angle/°	Magnitudes/MPa	Azimuth angle/°		
A	8.41	S89.33°E	2.54	S2.34°E	4.72	1.78
B	8.66	S87.07°E	3.25	S5.09°E	4.91	1.76

In this situation, the maximum principal horizontal stress was perpendicular to roadways along a North–South direction and parallel to roadways along an East–West direction. When the stress acted perpendicular to the roadway, it would induce much more damage than those stresses parallel to it. This was confirmed by large deformation (19.3 mm/day of two sides moving inward, and total amount 596 mm in monitoring period) in the return air cross-cut, and small deformation in the return airway in the first month after roadway excavation and support installation. About one month later, because of large swelling pressure in this extremely weak rock, the return airway also suffered large deformation (12.5 mm/day of two sides moving inward, and total amount 426 mm in monitoring period).

Under the influence of extremely weak rock, ground stress and swelling pressures, the roadway deformed to a significant extent, in which floor heave was particularly serious. Steel sets or bolts alone had little effect in controlling roadway deformation. So, a variety of supporting methods, such as closed 36U-shape steel sets and bolting, a pair of 12I-beam steel sets and bolting, were tested in situ. Although offering significant support strength, roadways deformed continuously and those supports failed to achieve an ideal effect. Because of the presence of expansive clay in this extremely weak rock, grouting was unfeasible. So, the alternative was to improve the strength of steel sets, or to improve the strength of bolts, or to improve the strength of both. This project mainly researched the effect of bolting in this extremely weak rock, included assessment of effect of roof and both sides bolting, and the effect of floor bolting.

## Methods

**Fig. 6:**
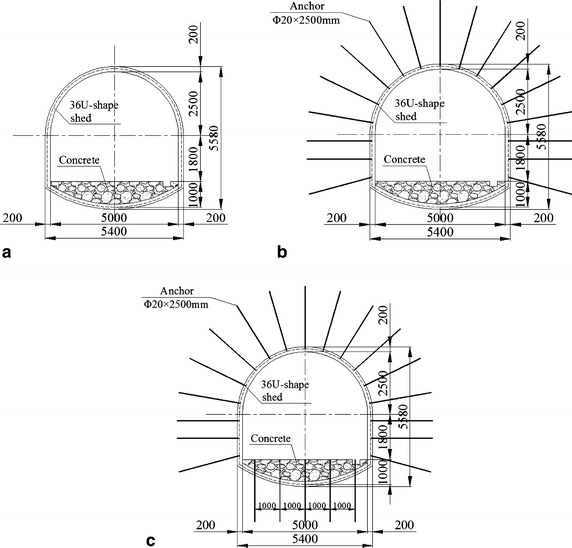
Roadway supporting approaches in prototype (unit: mm). **a** Model 1. **b** Model 2. **c** Model 3

In this extremely weak rock, because of large deformation (38.3 mm/day of floor heaving up, and total amount 1658 mm in monitoring period) occurring over a short time when unsupported, based on return air cross-cut deformations, three models with differnet support schemes were constructed and tested. In Model 1 (Fig. 6a), roadway was supported by 36U-shape steel sets. In Model 2 (Fig. 6b), roadway was supported by 36U-shape steel sets and bolting in its roof and two sides. In Model 3 (Fig. 6c), roadway was supported by 36U-shape steel sets and bolting in its roof, two sides, and floor. The effect of bolting in the roof and two sides was analysed by comparing roadway displacements in Models 1 and 2. The effect of floor bolting was analysed by comparing displacements in Models 2 and 3.

In prototype of Models 1 and 2, the type of anchor used in roof and two sides was Φ 20 × 2500 mm, full-size grouted. Closed 36U-shape steel set was installed in every 700 mm. At each 700 mm centre, a row of bolts was installed. In every row, each two bolts were at 700 mm in spacing (i.e. a row of bolts was installed between each pair of steel sets). The closed steel set had 1000 mm invert, which was filled with concrete.

### Ratio between prototype and model

Physical tests are commonly used in geological mechanics analysis. Similarity theorem indicats that model’s each element is similar to prototype’s corresponding element. Comprised by similar elements, the field and physical phenomenon between model and prototype are similar to each other. Based on similar physical phenomenon, the mechanics analysis in prototype can be deduced by model tests.

According to similarity theorems, as in prototype, material in model should be complied with Hooke’s law. Stress state of all points in the model should satisfy equilibrium equation, compatibility equation and geometric equation simultaneously. So the ratio (Yuan 1998) between prototype and model were defined as

Geometry ratio:

1 $$C_{l} = \frac{{l_{p} }}{{l_{m} }}$$

Volume-weight ratio:

2 $$C_{\gamma } = \frac{{\gamma_{p} }}{{\gamma_{m} }}$$

Stress ratio:

3 $$C_{\sigma } = C_{\gamma } \times C_{l}$$

Elasticity modulus ratio:

4 $$C_{E} = \frac{{E_{p} }}{{E_{m} }} = C_{\sigma }$$

Displacement ratio:

5 $$C_{\delta } = \frac{{\delta_{p} }}{{\delta_{m} }}$$

In which, *l*_*p*_, *γ*_*p*_, *E*_*p*_, *δ*_*p*_ were the size, volume-weight, elasticity modulus and displacement in prototype, respectively. *l*_*m*_, *γ*_*m*_, *E*_*m*_, *δ*_*m*_ were the size, volume-weight, elasticity modulus and displacement in model, respectively.

**Fig. 7:**
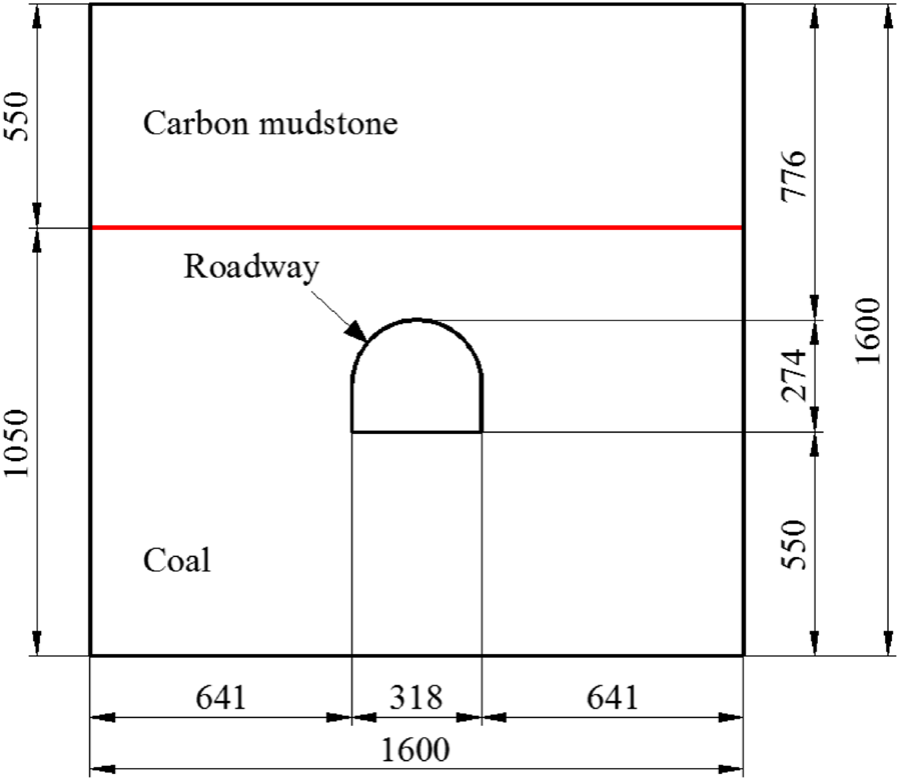
Model design

In the laboratory, the model frame size length, width, and height were 1600, 400, and 1600 mm respectively. According to site conditions, the roadway was 5000 mm wide and 4300 mm high. Considering the size of the influence zone of mining operations, the geometry ratio *C*_*l*_ between prototype and model was set as 16. According to the ratio, the roadway in model was 313 mm wide and 269 mm high. The steel set thickness, and exposed anchors on the roadway surface, meant that the model roadway size was enlarged by 5 mm in both width and height (i.e. the model roadway was 318 mm wide and 274 mm high). The roadway was designed to sit close to the centre of model (Fig. 7). In the model, the coal seam height was 1050 mm. To simplify the model, the rest of its height (550 mm) was designed as being carbon mudstone (it should be carbon mudstone and the 2^1^ coal seam as shown in Fig. 1).

### Materials

**Fig. 8:**
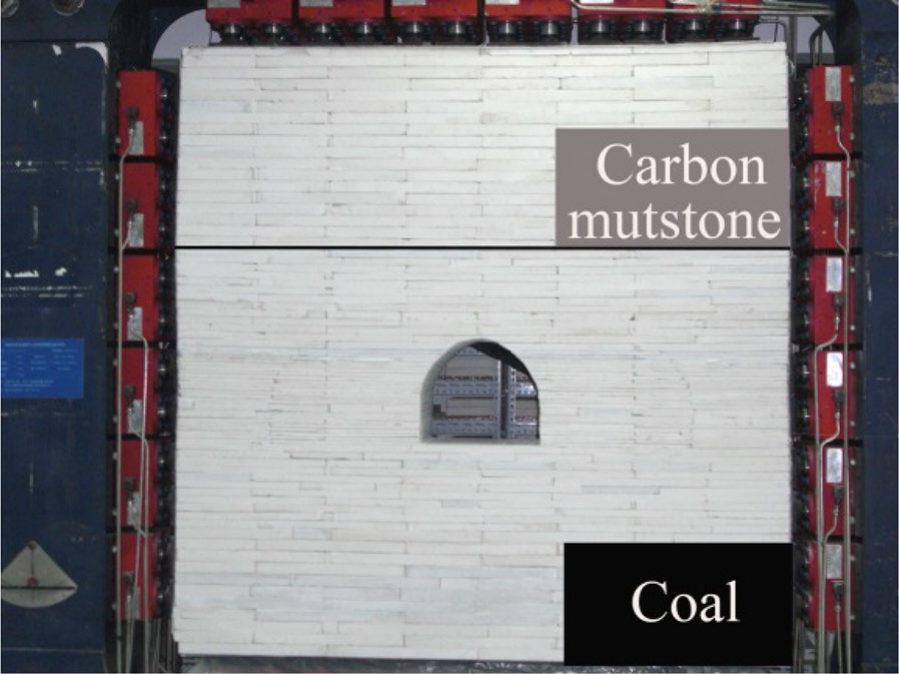
The paved model

From the mechanical parameters listed in Table 1, we determined the physical strata properties mainly based on average values of bulk density, compressive strength, tensile strength, and apparent cohesion. *C*_*γ*_ was found to be 1.176, *C*_l_ was 16. Correspondingly, *C*_*σ*_ was 18.82. Between carbon mudstone and coal seam strata, parameter of bulk density was in the ratio *C*_*γ*_, as well as parameters of compressive strength, tensile strength, and apparent cohesion were in the ratio *C*_*σ*_. According to the ratio, Carbon mudstone strata were gained by gypsum reacted with water, as well as the coal seam was formed by gypsum, water and polystyrene foam. Physical strata were found after repeated testing. There was no expansive characteristic in physical strata. The swelling force was applied by loading (more details in section of *Loading process*). Rock layers were paved (layer-by-layer) to simulate bedded deposit in situ. The paved model, before support installation, is shown in Fig. 8.

**Fig. 9:**
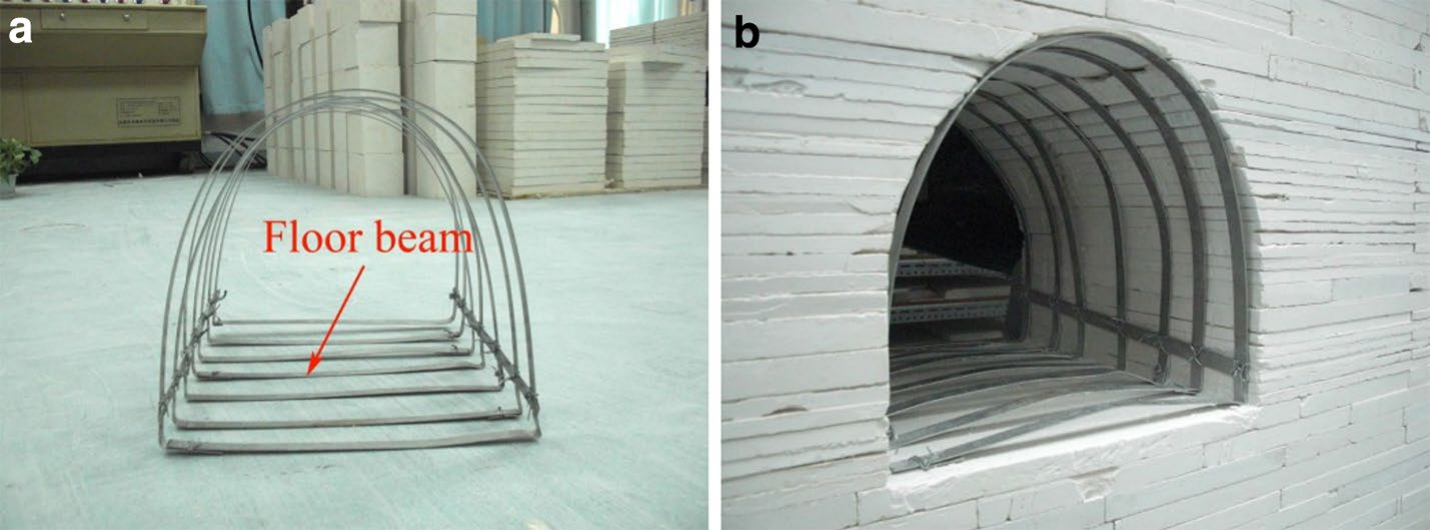
Model U-shape steel sets. **a** Steel sets. **b** Steel sets in roadway

To compare stiffness and deformation characteristics in different materials, steel bar (10 mm wide and 2 mm thick) was chosen to form the 36U-shape steel sets (Fig. 9a). According to the geometry ratio *C*_*l*_, the distance from steel set to set was 700 mm in situ and 44 mm in model. In prototype, there was a 1000 mm invert in the floor: when the invert was excavated, less influence arose therefrom in situ, but it would damage the integrity of the gypsum-composite floor in these tests. To preserve the integrity of the model floor, there was no excavation therein and the steel set invert was simplified to form a floor beam in models (Fig. 9a, b).

**Fig. 10:**
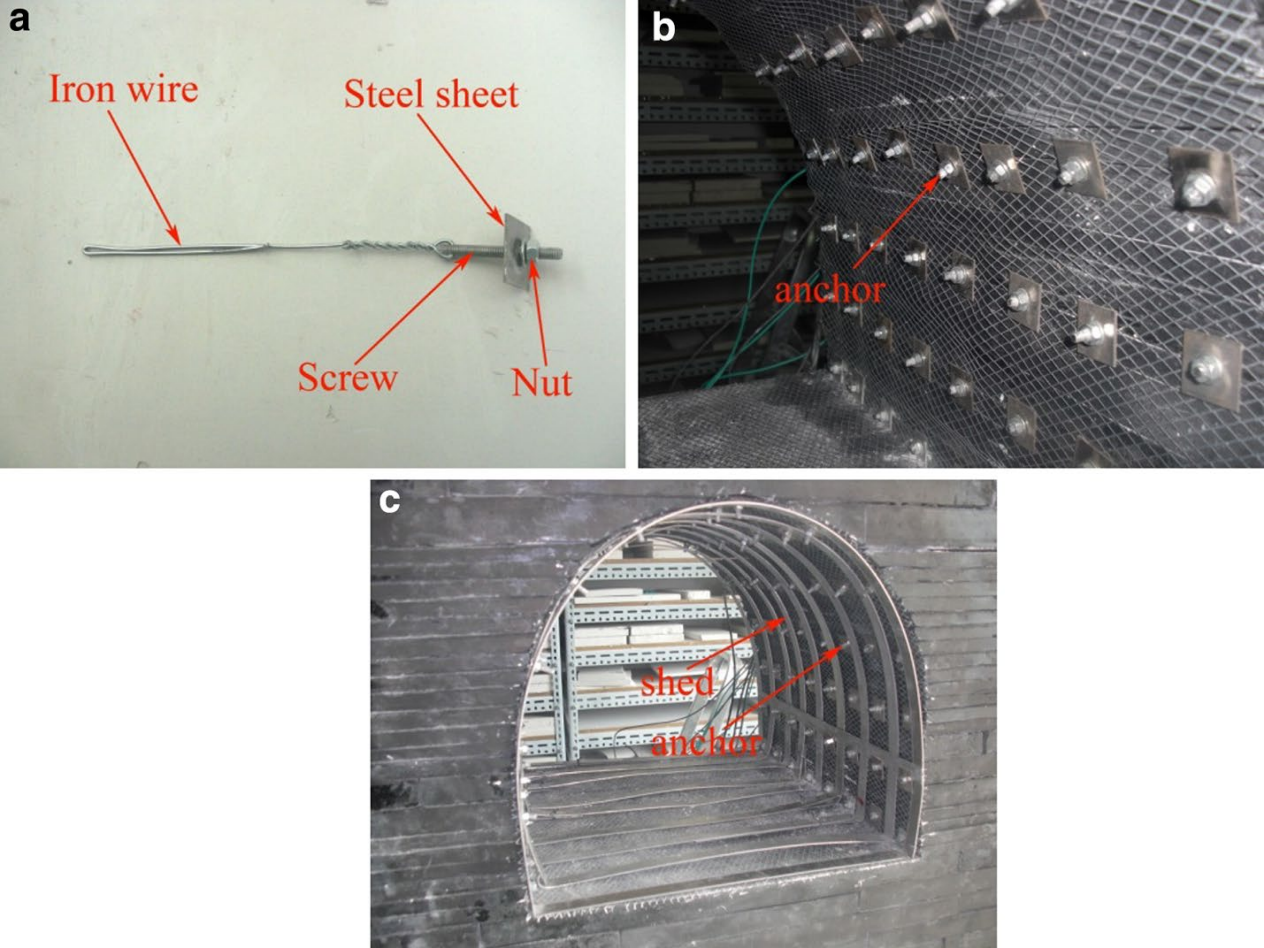
Model anchors. **a** Anchor. **b** Anchors in roadway. **c** Anchors and steel sets in roadway

Each anchor was composed of a rod, nut, and end plate. In model, each rod was made of Φ 4 × 40 mm screw and Φ 2 × 120 mm iron wire. There were holes at the end of each screw. Iron wire was connected to the end of screw by being threaded through the hole. The total length of each rod was 160 mm. The nut matching each screw was used as an anchor nut and 10 mm × 10 mm × 1 mm steel sheet was used for anchor plate. AB adhesive (an epoxy resin) was used as the anchoring agent. There was a fine metal mesh installed between the rock and the anchor plates (Fig. 10).

In each model, there were four displacement meters *S*_1_, *S*_2_, *S*_3_ and *S*_4_ for deformation measurement, which were set in roof (*S*_3_), floor (*S*_1_), and both sides (*S*_2_ and *S*_4_), respectively.

### Loading process

In NMC, the ground stress results (Table 2) indicated that: *σ*_*h*max_ was 8.41–8.66 MPa and *σ*_*v*_ was 4.72–4.91 MPa. To facilitate loading in the experiments, *σ*_*h*max_ was set to 8.6 MPa and *σ*_*v*_ was set to 4.8 MPa. Correspondingly, *σ*_*h*max_/*σ*_*v*_ was 1.8. According to *C*_*σ*_ being 18.82, *σ*_*h*max_ was 0.457 MPa and *σ*_*v*_ was 0.255 MPa in these tests. During loading, horizontal and vertical stresses were increased simultaneously. The vertical stress was increased in 0.1 MPa increments every 30 min: the horizontal stress was taken as the vertical stress multiplied by 1.8. When the vertical stress reached 0.3 MPa, the horizontal stress was 0.54 MPa: there were no signs of failure in surrounding rock. Accordingly, ground stress was not the cause of roadway deformation in this extremely weak rock. This weak, fractured rock was easily weathered, prone to disintegration, and easily swollen. So the next loading stage was changed to reflect the influence of swelling. Under the same excavation conditions, the swelling pressure in the surrounding rock was almost equal everywhere. In view of this, and based on existing loads, the vertical and horizontal stresses increased simultaneously in 0.1 MPa increments every 30 min. The loading was not stopped until the model was damaged. Loading regimes in Models 1–3 are shown in Tables 3, 4 and 5, respectively.

**Table 3:** Loading grades in Model 1 (MPa)

Grade	Vertical load	Side load	Grade	Vertical load	Side load
1st	0.1	0.18	5th	0.5	0.74
2nd	0.2	0.36	6th	0.6	0.84
3rd	0.3	0.54	7th	0.7	0.94
4th	0.4	0.64	8th	0.8	1.04

**Table 4:** Loading grades in Model 2 (MPa)

Grade	Vertical load	Side load	Grade	Vertical load	Side load
1st	0.1	0.18	7th	0.7	0.94
2nd	0.2	0.36	8th	0.8	1.04
3rd	0.3	0.54	9th	0.9	1.14
4th	0.4	0.64	10th	1.0	1.24
5th	0.5	0.74	11th	1.1	1.34
6th	0.6	0.84	12th	1.2	1.44

**Table 5:** Loading grades in Model 3 (MPa)

Grade	Vertical load	Side load	Grade	Vertical load	Side load
1st	0.1	0.18	8th	0.8	1.04
2nd	0.2	0.36	9th	0.9	1.14
3rd	0.3	0.54	10th	1.0	1.24
4th	0.4	0.64	11th	1.1	1.34
5th	0.5	0.74	12th	1.2	1.44
6th	0.6	0.84	13th	1.3	1.54
7th	0.7	0.94	14th	1.4	1.64

## Results

Under different support regimes, the roadway underwent different amounts of deformation so the efficacy of each set of supporting conditions could be quantified on the basis of their relative displacements. Taking the roof as an example, it was assumed that the roof subsidence was *D*_0_ under original support conditions and *D*_1_ under a new support regime. In this situtation, the reinforcing coefficient *Q* of new supporting on roof can be defined as:

6 $$Q = \left| {\frac{{D_{0} }}{{D_{1} }}} \right|$$

The reinforcing coefficient on two sides and the floor were treated in the same way. Displacements of the floor, right side, roof, and left side can be obtained by measurements at *S*_1_, *S*_2_, *S*_3_, and *S*_4_. Two sides displacements were almost the same, so left side displacement was used in this analysis only. When *Q* > 1, it is indicated that the new supports could control roadway deformation. The larger the value of *Q*, the better the new supports in deformation control. When *Q* < 1, it is indicated that the new supports could not control roadway deformation.

### Analysis of bolting in the roof and on two sides

Before the eighth load increment was applied to Model 1, the displacements of roof, two sides and floor reached 19.0, 22.8 and 36.0 mm, respectively. And before the twelfth load increment was applied to Model 2, the displacements of roof, two sides and floor reached 17.8, 14.3 and 29.0 mm, respectively. Under these two conditions, the roadway deformed to a significant extent, but remained stable. When the eighth load increment was applied to Model 1, and the twelfth to Model 2, the roadway deformation increased and failure ensued soon after. The displacements here were so large as to be meaningless when analysing the role of any supports, so displacements before these stages were used in subsequent analysis.

**Fig. 11:**
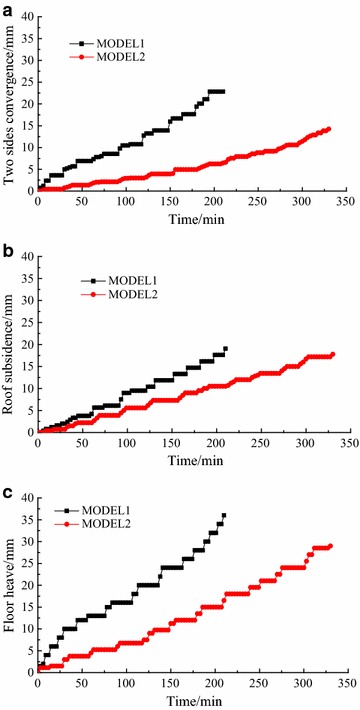
Displacements during loading: Models 1 and 2. **a** Two sides deformation. **b** Roof deformation. **c** Floor deformation

**Fig. 12:**
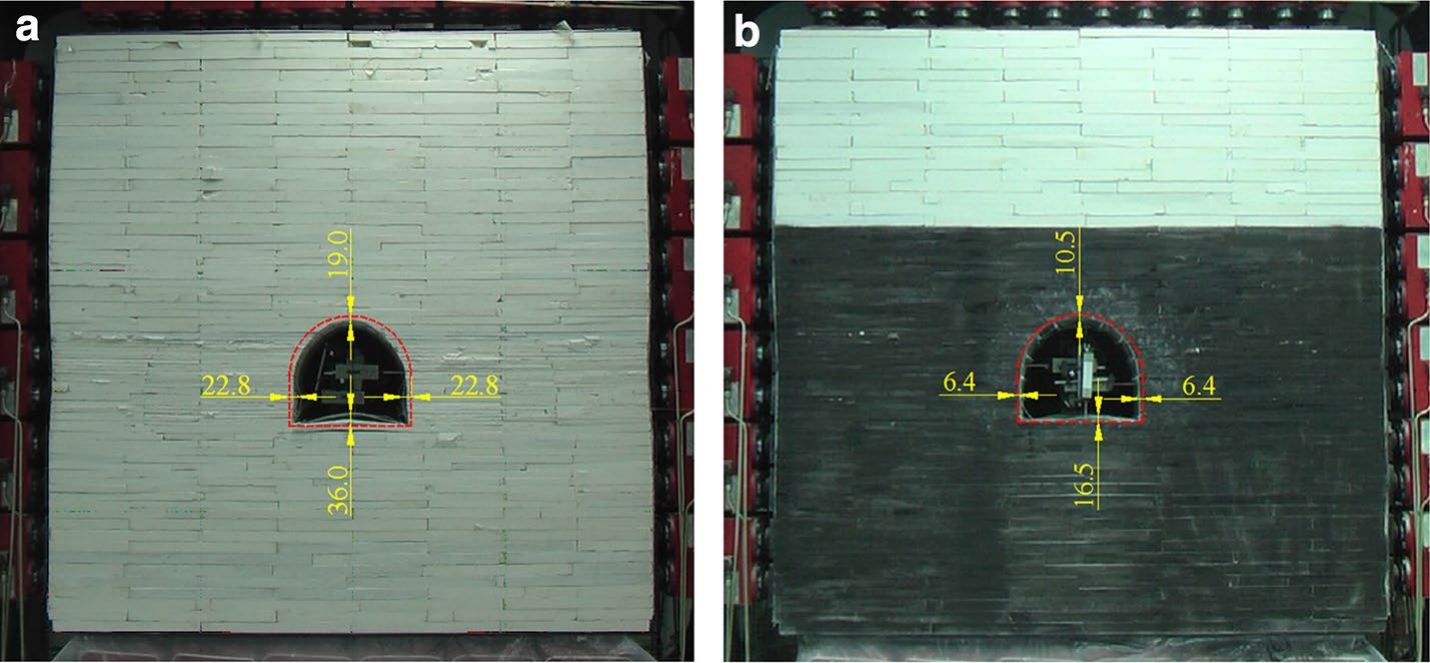
Roadway displacements after seventh load increment. **a** Displacements in Model 1. **b** Displacements in Model 2

The relationships between displacements and loading times are shown in Fig. 11: after bolting, under the same load, the deformations at the same places decreased. When the seventh load increment was completed, displacements of the roof, left side, and floor 19.0, 22.8, and 36.0 mm in Model 1, but 10.5, 6.4, and 16.5 mm in Model 2 (Fig. 12; Table 6). The bolting used on roof and two sides showed significant role in side-reinforcement, and relatively small role in roof strengthening. According to (6), the reinforcing coefficient of bolting in roof and two sides reach 3.56 on two sides, and 1.81 on roof: because of the integral structure of the surrounding rock, bolting also played a role in strengthening the floor. Although no bolting was used in floor, the reinforcing coefficient on floor reached 2.18, showing a greater effect than on roof.

**Table 6:** Roadway displacements upon completion of the seventh load increment

Model	Roof subsidence/mm	Left side convergence/mm	Right side convergence/mm	Floor heave/mm
1	19.0	22.8	22.8	36.0
2	10.5	6.4	6.4	16.5
Reinforcing coefficient	1.81	3.56	3.56	2.18

### Analysis of floor bolting

Before the twelfth load increment in Model 2, and the fourteenth in Model 3, the roadway remained stable. Thereafter the roadways were damaged within a few minutes: displacements before the twelfth load increment in Model 2, and the fourteenth in Model 3 were analysed.

**Fig. 13:**
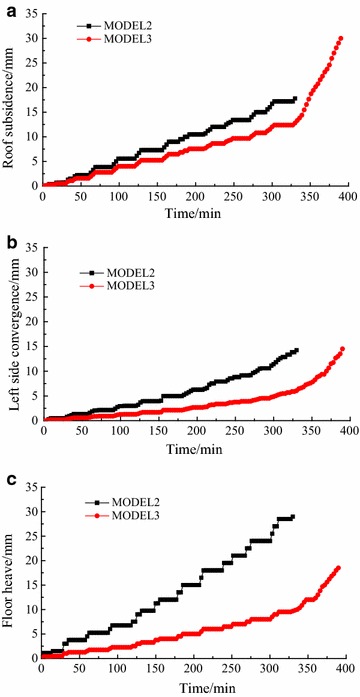
Displacements during loading: Models 2 and 3. **a** Roof deformation. **b** Left side deformation. **c** Floor deformation

**Fig. 14:**
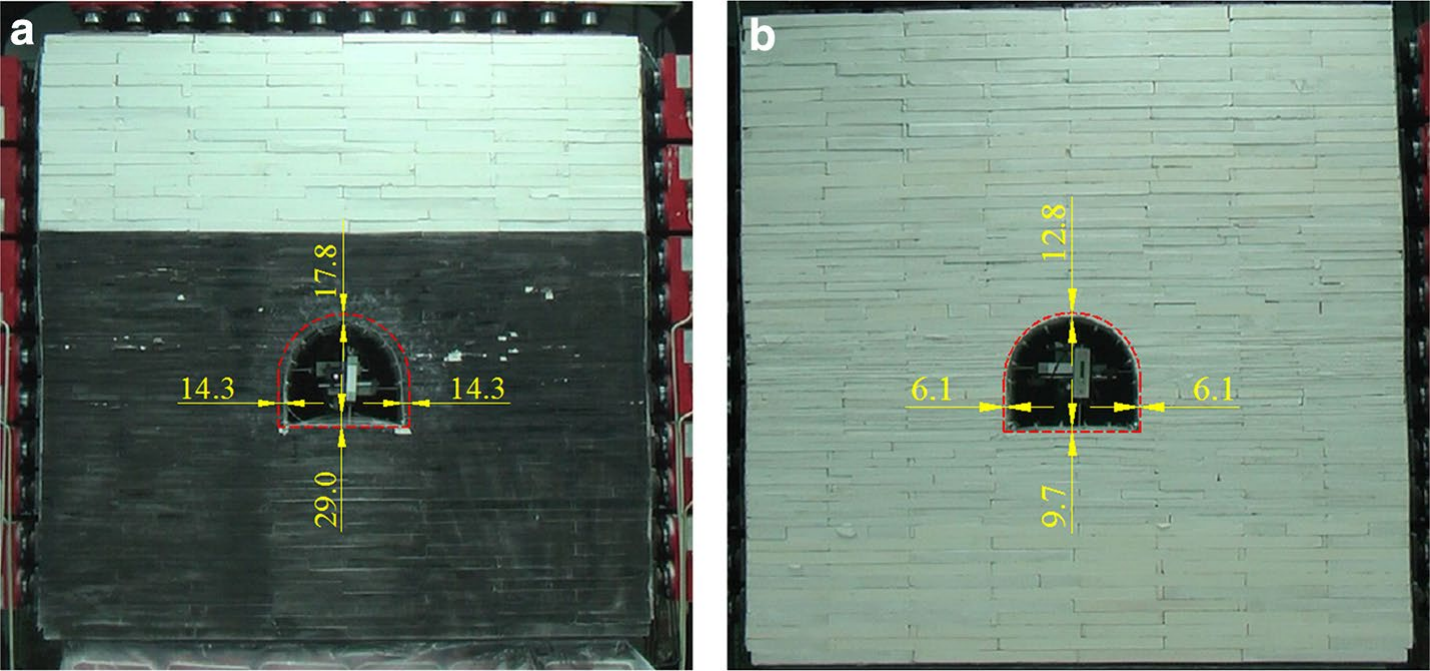
Roadway displacements after eleventh load increment. **a** Displacements in Model 2. **b** Displacements in Model 3

Relationships between displacements and loading times are shown in Fig. 13: after floor bolting was added, under the same load, deformation at each point decreased. When the eleventh load increment was completed, displacements of roof, left side, and floor were 17.8, 14.3, and 29.0 mm in Model 2, but 12.8, 6.1, and 9.7 mm in Model 3 (Fig. 14; Table 7). According to (6), the floor bolting reinforcing coefficient on floor was 3.06. Correspondingly, the reinforcing coefficient was 2.34 on two sides and 1.39 on roof. Floor bolting, therefore, had the greatest effect on floor reinforcement, a larger effect on two sides, and a large effect on roof. The effect on floor was more than twice that on roof. The floor was closer to sides than to roof, so it was thought that the effect of floor bolting decreased as distance to floor increased. So in these similar geological conditions, floor bolting was an effective technique in floor heave control.

**Table 7:** Roadway displacements upon completion of the 12th load increment

Model	Roof subsidence/mm	Left side convergence/mm	Right side convergence/mm	Floor heave/mm
2	17.8	14.3	14.3	29.0
3	12.8	6.1	6.1	9.7
Reinforcing coefficient	1.39	2.34	2.34	3.06

**Fig. 15:**
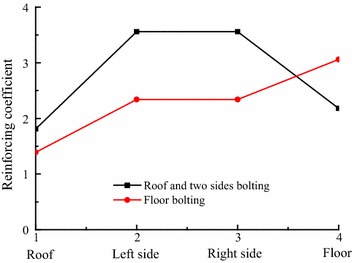
Roadway reinforcing coefficients (four locations)

The data show that roof and sides bolting had the biggest effect in reinforcing two sides, bigger effect in floor strengthening, and big effect in roof controlling (Fig. 15). The reinforcing coefficients on two sides, floor, and roof reached 3.56, 2.18, and 1.81 respectively. Based on bolting in roof and two sides, floor bolting had the biggest effect in floor reinforcing, bigger effect in two sides strengthening, and big effect in roof controlling (Fig. 15). The reinforcing coefficients on floor, two sides, and roof reached 3.06, 2.34, and 1.39 respectively. So the overall effect on surrounding rock was remarkable. When roof and sides were reinforced, the floor heave decreased. Correspondingly, when floor strength was improved, convergence of two sides was better controlled. So in this extremely weak rock, the surrounding rock should be considered as an integral in supporting, which will achieve better effect than local strength improvement, especially in areas adjacent to two sides and floor.

## Conclusions

The surrounding rock formed an integral structure: when one area was reinforced, other areas were affected. Based on extremely weak rock in NMC, three physical models were produced and tested in laboratory. Through physical simulation, this research quantified the effect of bolting as a roadway support mechanism.

The results indicated that the reinforcing coefficients of bolting in roof and sides, on floor, two sides and roof, reached 2.18, 3.56, and 1.81 respectively. The reinforcing coefficient of floor bolting, on floor, two sides and roof reached 3.06, 2.34, and 1.39 respectively. The data show that roof and sides bolting had the biggest effect in reinforcing two sides, bigger effect in floor strengthening, and big effect in roof controlling. Although no bolting was used in floor, the reinforcing coefficient on floor reached 2.18, showing a greater effect than on roof. Based on bolting in roof and two sides, floor bolting had the biggest effect in floor reinforcing, bigger effect in two sides strengthening, and big effect in roof controlling, which indicated that the effect of floor bolting decreased as distance to floor increased.

From above we could get that the overall effect on surrounding rock was remarkable. When roof and sides were reinforced, the floor heave decreased, and when floor strength was improved, convergence of two sides was better controlled. So in this extremely weak rock, surrounding rock should be considered as an entire, integral structure, when designing a support scheme: this will lead to better results than local strength improvement.
